# Free Vibration Analysis of a Graphene-Platelet-Reinforced, Porous, Two-Cylindrical-Panel System

**DOI:** 10.3390/ma15176158

**Published:** 2022-09-05

**Authors:** Xianguang Sun, Weichao Chi, Jia Luo

**Affiliations:** 1 Key Laboratory of Structural Dynamics of Liaoning Province, College of Sciences, Northeastern University, Shenyang 110819, China; 2 Beijing Institute of Structure and Environment Engineering, Beijing 100076, China

**Keywords:** double-cylindrical-panel system, graphene platelets, metal foam, free vibration, Chebyshev polynomials

## Abstract

In this study, a novel, dynamic model of a graphene-platelet-reinforced, porous (GPLRP) double-cylindrical-panel system is proposed. The material properties of a graphene-platelet-reinforced, porous, double-cylindrical-panel system were determined by the Halpin–Tsai micromechanics model and the typical mechanical properties of open-cell metal foams. Different types of porosity distribution and graphene platelet (GPL) distribution patterns were considered. Love’s shell theory was utilized to derive the theoretical formulation, and the Rayleigh–Ritz method was used to calculate the natural frequencies of the system. The proposed model was validated by several comparison studies with the natural frequencies in the existing literature. Finally, the effects of stiffness of Winkler springs, boundary condition, porosity coefficient, porosity distribution, GPL distribution pattern, and GPL weight fraction on the free vibration characteristics of the system were evaluated.

## 1. Introduction

The two-cylindrical-panel system, an important component of modern lightweight structures, is widely used in mechanical, vehicle, and marine structures [1,2]. A two-cylindrical-panel system may possess ideal structural properties, such as better vibration attenuation and lighter weight, than a single cylindrical panel [3,4]. Therefore, the in-depth study of such systems has great theoretical and practical significance.

Recently, research on the vibration characteristics of multi-beam systems and multi-plate systems has been of great interest [5,6,7,8,9,10,11,12]. Many experts have conducted a large number of studies on the vibration characteristics of multi-beam systems [13,14,15,16,17,18]. Kim et al. [19,20] established a double-beam system and analyzed the free vibration characteristics of a system. Deng et al. [21] studied the vibration characteristics of a double-functionally-graded (FG) beam system on an elastic foundation. Hao et al. [22] utilized the modified Fourier–Ritz method to analyze the free vibration characteristics of a double-beam system. Rahman and Lee [23] developed a novel harmonic balance method to analyze the nonlinear vibration characteristics of a double-beam system. Kelly and Srinivas [24] established a system of multiple beams connected by elastic layers and investigated the free vibration of the system. Han et al. [25] discussed the vibration characteristics of a double-beam system by using the improved Wittrick–Williams algorithm.

Many researchers have also conducted numerous studies on the vibration characteristics of a multi-plate system. Oniszczuk [26] investigated the free vibration of two plates connected by a Winkler elastic layer. Based on the Rayleigh–Ritz method, Jeong and Kang [27] developed a novel theoretical method to study the vibration characteristics of multiple rectangular plates coupled by a liquid. Hedrih [28,29] investigated the free vibration characteristics of double-plate systems. Stojanović et al. [30] revealed the vibration and stability characteristics of a system of multiple rectangular plates connected with elastic layers by using an analytical method. The above studies on multi-plate structures are limited to rectangular plates, though many scholars have also carried out detailed studies on circular plates. Hedrih and Simonović [31,32,33] presented a dynamic model of the double circular plate system and investigated the non-linear characteristics of the system. Noga [34] established a dynamic model of double annular and circular membranes connected with the Winkler elastic layer and studied the free vibration of the system by using analytical and numerical methods.

Metal foam materials are widely employed in energy-absorbing systems due to their energy-absorbing capability [35] and other particular characteristics [36]. Keleshteri and Jelovica [37] investigated the free vibration characteristics of the FG porous cylindrical panels. Wang and Wu [38] analyzed the influences of the porous coefficient on the free vibration of an FG porous cylindrical shell.

Due to their pores, porous metal structures are light, but they also can reduce the stiffness of such a structure, so filling materials are needed to enhance the structural stiffness. GPLs possess good mechanical properties, which is why they are employed as reinforcement materials and widely used in various composite structures [39,40]. Using the GPLRP material, Yang et al. [41] analyzed the buckling and free vibration characteristics of a plate, and Wang et al. [42] studied the nonlinear free vibration characteristics of circular cylindrical shells. Chai and Wang [43] studied the traveling wave vibration characteristics of GPLRP-joined conical-cylindrical shells in a spinning motion. Ye and Wang [44] analyzed the nonlinear forced vibration of GPLRP cylindrical shells. Xu et al. [45] investigated the free vibration of a rotating GPLRP beam by using the differential transformation method. Teng and Wang [46] studied the primary, superharmonic, and subharmonic resonances of GPLRP plates. Zhou et al. [47] established an accurate nonlinear buckling analysis of the GPLRP composite cylindrical shells under an axial compressive load. Twinkle and Jeyaraj [48] analyzed the buckling and vibration characteristics of a GPLRP, cylindrical panel. The above studies indicate that a GPLRP structure has good vibration absorption performance and high strength, so it has good prospects for engineering structures.

The above review shows that most studies mainly focus on the vibration characteristics of plane systems, and there are almost no studies on curved systems. To fill the gap in the studies of curved systems, a novel dynamic model of a graphene-platelet-reinforced, porous (GPLRP) two-cylindrical-panel system based on Love’s shell theory is established. To validate the proposed model, several comparisons of the present results with those from open literature are made. Subsequently, the effects of stiffness of the Winkler springs, boundary condition, porosity coefficient, porosity distribution, GPL distribution pattern, and GPL weight fraction on the free vibration characteristics of the system are investigated using the Rayleigh–Ritz method.

## 2. Theoretical Formulations

### 2.1. Description of the Model

**Figure 1:**
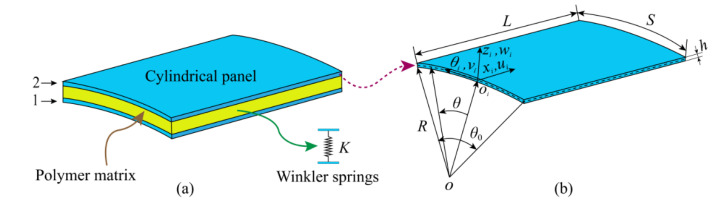
Schematic diagram of the GPLRP, two-cylindrical-panel system. (**a**) two-cylindrical-panel system; (**b**) single-cylindrical-panel.

The schematic diagram of the GPLRP, two-cylindrical-panel system with the radius of curvature *R*, thickness *h*, subtended angle *θ*_0_, axial length *L*, and arc length *S* (*S* = *Rθ*_0_) coupled by a polymer matrix is shown in Figure 1. As shown in Figure 1, the local cylindrical coordinate system (*x_i_*, *θ_i_*, *z_i_*) of the *i*th (*i* = 1, 2) GPLRP, cylindrical panel is established on the midplane of the GPLRP, cylindrical panel, the origin *o_i_* of which is located at the midpoint on the left side of the midplane. The displacements of the arbitrary point of the midplane are denoted by *u_i_*, *v_i_*, and *w_i_* along with the directions *x_i_*, *θ_i_*, and *z_i_*, respectively. The polymer matrix between the GPLRP double cylindrical panels can be considered equivalent to continuously distributed Winkler springs with stiffness *K.* Both of the GPLRP, cylindrical panels have identical material parameters, geometric parameters, and boundary conditions.

**Figure 2:**
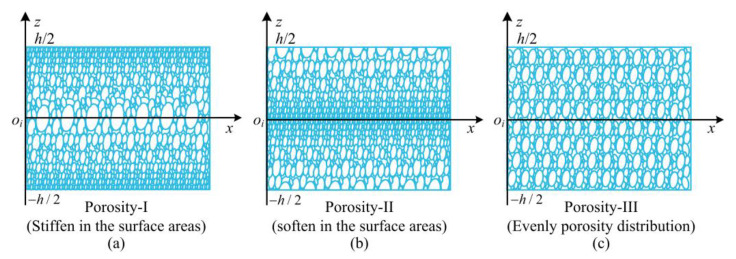
Cross-section of a single GPLRP, cylindrical panel for different porosity distributions. (**a**) Porosity-I; (**b**) Porosity-II; (**c**) Porosity-III.

As shown in Figure 2, three types of porosity distribution along the thickness direction, denoted by porosity-I, porosity-II, and porosity-III, are considered in this study. For different porosity distributions, the Young’s modulus, mass density, and Poisson’s ratio along the thickness direction of GPLRP, cylindrical panel can be given as follows [41]:

(1) $$E(z)=\{\begin{array}{ll} E^{*}[1-e_{1}\cos(\frac{\pi z}{h})] & \mathrm{Porosity}-Ι\\ E^{*}\{1-e_{2}[1-\cos(\frac{\pi z}{h})]\} & \mathrm{Porosity}-ΙΙ\\ E^{*}e_{3} & \mathrm{Porosity}-ΙΙΙ \end{array}$$

(2) $$\rho (z)=\{\begin{array}{ll} \rho^{*}[1-e_{m1}\cos(\frac{\pi z}{h})] & \mathrm{Porosity}-Ι\\ \rho^{*}\{1-e_{m2}[1-\cos(\frac{\pi z}{h})]\} & \mathrm{Porosity}-ΙΙ\\ \rho^{*}e_{m3} & \mathrm{Porosity}-ΙΙΙ \end{array}$$

(3) $$\mu (z)=\mu^{*}$$

in which E*, ρ*, and μ* represent Young’s modulus, mass density, and Poisson’s ratio of the GPL-reinforced cylindrical panel without pores, respectively. *e*_1_, *e*_2_, and *e*_3_ are the porosity coefficients; *e*_m1_, *e*_m2_, and *e*_m3_ are the mass density coefficients.

According to the Halpin–Tsai micromechanics model, the effective Young’s modulus of the GPL-reinforced cylindrical panel without porosities can be given as follows [41]:

(4) $$E^{*}=\frac{3}{8}\left[\frac{1+\xi_{L}^{\mathrm{GPL}}\eta_{L}^{\mathrm{GPL}}V_{\mathrm{GPL}}(z)}{1-\eta_{L}^{\mathrm{GPL}}V_{\mathrm{GPL}}(z)}\right]E_{m}+\frac{5}{8}\left[\frac{1+\xi_{w}^{\mathrm{GPL}}\eta_{w}^{\mathrm{GPL}}V_{\mathrm{GPL}}(z)}{1-\eta_{w}^{\mathrm{GPL}}V_{\mathrm{GPL}}(z)}\right]E_{m}$$

in which

(5) $$\xi_{L}^{\mathrm{GPL}}=\frac{2l_{\mathrm{GPL}}}{t_{\mathrm{GPL}}},\xi_{w}^{\mathrm{GPL}}=\frac{2w_{\mathrm{GPL}}}{t_{\mathrm{GPL}}}$$

(6) $$\eta_{L}^{\mathrm{GPL}}=\frac{E_{\mathrm{GPL}}-E_{m}}{E_{\mathrm{GPL}}+\xi_{L}^{\mathrm{GPL}}E_{m}},\eta_{w}^{\mathrm{GPL}}=\frac{E_{\mathrm{GPL}}-E_{m}}{E_{\mathrm{GPL}}+\xi_{w}^{\mathrm{GPL}}E_{m}}$$

where *E*_GPL_ and *E*_m_ represent Young’s moduli of the GPLs and metal matrix; *l*_GPL_, *w*_GPL_, and *t*_GPL_ represent the average length, width, and thickness of the GPLs, respectively; *V*_GPL_(*z*) is the volume fraction of the GPLs.

The mass density ρ* and Poisson’s ratio μ* of the GPL reinforced cylindrical panel without porosities can be defined by [41]

(7) $$\rho^{*}=\rho_{m}(1-V_{\mathrm{GPL}}(z))+\rho_{\mathrm{GPL}}V_{\mathrm{GPL}}(z)$$

(8) $$\mu^{*}=\mu_{m}(1-V_{\mathrm{GPL}}(z))+\mu_{\mathrm{GPL}}V_{\mathrm{GPL}}(z)$$

in which *ρ*_GPL_ and *μ*_GPL_ represent the mass density and Poisson’s ratio of the GPLs, respectively; *ρ*_m_ and *μ*_m_ denote the mass density and Poisson’s ratio of the metal matrix, respectively.

The relationship between the modulus of elasticity and mass density of the open-cell metal foams is as follows [41]:

(9) $$\frac{E(z)}{E^{*}}={\left(\frac{\rho (z)}{\rho^{*}}\right)}^{2}$$

The relationship between the mass density coefficients and the porosity coefficients can be obtained by substituting Equations (1) and (2) into Equation (9):

(10) $$\{\begin{array}{l} 1-e_{m1}\cos(\pi z/h)=\sqrt{1-e_{1}\cos(\pi z/h)}\\ 1-e_{m2}[1-\cos(\pi z/h)]=\sqrt{1-e_{2}[1-\cos(\pi z/h)]}\\ e_{m3}=\sqrt{e_{3}} \end{array}$$

To obtain a valuable comparison of different combinations, the total mass of the GPLRP, cylindrical panel was set to be the same for various types of porosity and GPL distribution, resulting in

(11) $$\int_{0}^{h/2}\sqrt{1-e_{1}\cos(\pi z/h)}dz=\int_{0}^{h/2}\sqrt{1-e_{2}[1-\cos(\pi z/h)]}dz=\int_{0}^{h/2}\sqrt{e_{3}}dz$$

in which *e*_2_ and *e*_3_ can be determined by *e*_1_.

**Figure 3:**
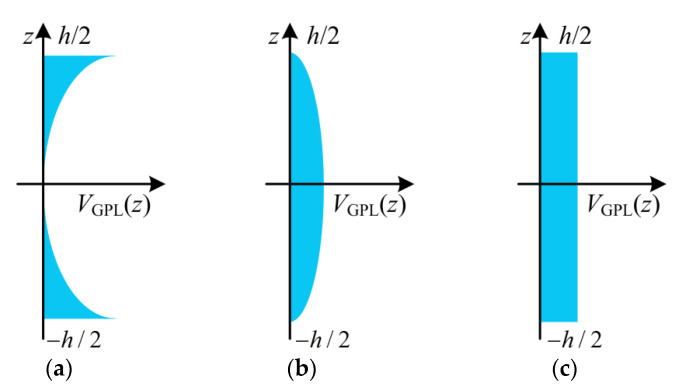
Three types of GPL distribution patterns. (**a**) GPL A; (**b**) GPL B; (**c**) GPL C.

To reinforce the porous cylindrical panel, the GPLs are applied as fillers inside the porous materials. The three GPL distribution patterns in the thickness direction of the cylindrical panel are shown in Figure 3. The volume fraction *V*_GPL_(*z*) of the GPLs can be written as follows [41]:

(12) $$V_{\mathrm{GPL}}(z)=\{\begin{array}{ll} s_{j1}[1-\cos(\pi z/h)] & \mathrm{GPL}A\\ s_{j2}\cos(\pi z/h) & \mathrm{GPL}B\\ s_{j3} & \mathrm{GPL}C \end{array}$$

in which *s_j_*_1_, *s_j_*_2_, and *s_j_*_3_ are the coefficients of the GPL volume fraction for different porosity distributions and GPL distribution patterns, and *j* = 1, 2, 3 correspond to the Porosity-I, -II, and -III distributions, respectively. The *s_j_*_1_, *s_j_*_2_, and *s_j_*_3_ can be given as follows [41]:

(13) $$V_{\mathrm{GPL}}^{T}\int_{-h/2}^{h/2}\frac{\rho (z)}{\rho^{*}}dz=\{\begin{array}{l} s_{j1}\int_{-h/2}^{h/2}[1-\cos(\pi z/h)]\frac{\rho (z)}{\rho^{*}}dz\\ s_{j2}\int_{-h/2}^{h/2}\cos(\pi z/h)\frac{\rho (z)}{\rho^{*}}dz\\ s_{j3}\int_{-h/2}^{h/2}\frac{\rho (z)}{\rho^{*}}dz \end{array}$$

where $V_{\mathrm{GPL}}^{T}$ is the total volume fraction of the GPLs and given by [41]

(14) $$V_{\mathrm{GPL}}^{T}=\frac{W_{\mathrm{GPL}}}{W_{\mathrm{GPL}}+(\rho_{\mathrm{GPL}}/\rho_{m})(1-W_{\mathrm{GPL}})}$$

where *W*_GPL_ is the weight fraction obtained by the ratio of the mass of the GPL to the mass of the cylindrical panel.

### 2.2. Governing Equations and Solution

According to Love’s shell theory [49], the strains of the arbitrary point at the distance *z* from the midplane can be written as

(15) $$\left\{\begin{array}{c} \varepsilon_{x}^{\left(i\right)}\\ \varepsilon_{\theta }^{\left(i\right)}\\ \gamma_{x\theta }^{\left(i\right)} \end{array}\right\}=\left\{\begin{array}{c} \varepsilon_{1}^{\left(i\right)}\\ \varepsilon_{2}^{\left(i\right)}\\ \gamma_{12}^{\left(i\right)} \end{array}\right\}+z\left\{\begin{array}{c} \varphi_{1}^{\left(i\right)}\\ \varphi_{2}^{\left(i\right)}\\ 2\varphi_{12}^{\left(i\right)} \end{array}\right\},\left(i=1,2\right)$$

where, $\varepsilon_{1}^{\left(i\right)}$,$\varepsilon_{2}^{\left(i\right)}$, and $\gamma_{12}^{\left(i\right)}$; and $\varphi_{1}^{\left(i\right)}$, $\varphi_{2}^{\left(i\right)}$, and $\varphi_{12}^{\left(i\right)}$ are midplane strains and surface curvatures of the *i*th GPLRP, cylindrical panel, which can be expressed by

(16) $$\varepsilon_{1}^{\left(i\right)}=\frac{\partial u_{i}}{\partial x_{i}},\varepsilon_{2}^{\left(i\right)}=\frac{1}{R}\left(\frac{\partial v_{i}}{\partial \theta_{i}}+w_{i}\right),\gamma_{12}^{\left(i\right)}=\frac{\partial v_{i}}{\partial x_{i}}+\frac{1}{R}\frac{\partial u_{i}}{\partial \theta_{i}}$$

(17) $$\varphi_{1}^{\left(i\right)}=-\frac{\partial^{2}w_{i}}{\partial x_{i}{}^{2}},\varphi_{2}^{\left(i\right)}=-\frac{1}{R^{2}}\left(\frac{\partial^{2}w_{i}}{\partial \theta_{i}{}^{2}}-\frac{\partial v_{i}}{\partial \theta_{i}}\right),\varphi_{12}^{\left(i\right)}=-\frac{1}{R}\left(\frac{\partial^{2}w_{i}}{\partial x_{i}\partial \theta_{i}}-\frac{\partial v_{i}}{\partial x_{i}}\right)$$

The stress–strain relationship of the *i*th GPLRP, cylindrical panel can be defined as [50]

(18) $$\left\{\begin{array}{c} \sigma_{x}^{\left(i\right)}\\ \sigma_{\theta }^{\left(i\right)}\\ \tau_{x\theta }^{\left(i\right)} \end{array}\right\}=\left[\begin{array}{ccc} Q_{11}^{\left(i\right)} & Q_{12}^{\left(i\right)} & 0\\ Q_{12}^{\left(i\right)} & Q_{22}^{\left(i\right)} & 0\\ 0 & 0 & Q_{66}^{\left(i\right)} \end{array}\right]\left\{\begin{array}{c} \varepsilon_{x}^{\left(i\right)}\\ \varepsilon_{\theta }^{\left(i\right)}\\ \gamma_{x\theta }^{\left(i\right)} \end{array}\right\}$$

where

(19) $$Q_{11}^{\left(i\right)}=Q_{22}^{\left(i\right)}=\frac{E(z)}{1-\mu (z)\mu (z)},Q_{12}^{\left(i\right)}=\frac{\mu (z)E(z)}{1-\mu (z)\mu (z)},Q_{66}^{\left(i\right)}=\frac{E(z)}{2\left(1+\mu (z)\right)}$$

The force and moment resultants of the *i*th GPLRP, cylindrical panel can be obtained by integrating the stresses along with the *z*-direction of the GPLRP, cylindrical panel, which are expressed as

(20) $$\left[\begin{array}{c} N_{x}^{\left(i\right)}\\ N_{\theta }^{\left(i\right)}\\ N_{x\theta }^{\left(i\right)} \end{array}\right]=\int_{-\frac{h}{2}}^{\frac{h}{2}}\left[\begin{array}{c} \sigma_{x}^{\left(i\right)}\\ \sigma_{\theta }^{\left(i\right)}\\ \tau_{x\theta }^{\left(i\right)} \end{array}\right]dz$$

(21) $$\left[\begin{array}{c} M_{x}^{\left(i\right)}\\ M_{\theta }^{\left(i\right)}\\ M_{x\theta }^{\left(i\right)} \end{array}\right]=\int_{-\frac{h}{2}}^{\frac{h}{2}}\left[\begin{array}{c} \sigma_{x}^{\left(i\right)}\\ \sigma_{\theta }^{\left(i\right)}\\ \tau_{x\theta }^{\left(i\right)} \end{array}\right]zdz$$

By substituting Equations (15) and (18) into Equations (20) and (21), we get:

(22) $$N_{\left(i\right)}^{T}=\varepsilon_{\left(i\right)}^{T}\cdot S^{\left(i\right)}$$

where $\varepsilon_{\left(i\right)}^{T}$ and $N_{\left(i\right)}^{T}$ are given by

(23) $$\begin{array}{l} \varepsilon_{\left(i\right)}^{T}=\left\{\varepsilon_{1}^{\left(i\right)},\varepsilon_{2}^{\left(i\right)},\gamma_{12}^{\left(i\right)},\varphi_{1}^{\left(i\right)},\varphi_{2}^{\left(i\right)},2\varphi_{12}^{\left(i\right)}\right\}\\ N_{\left(i\right)}^{T}=\left\{N_{x}^{\left(i\right)},N_{\theta }^{\left(i\right)},N_{x\theta }^{\left(i\right)},M_{x}^{\left(i\right)},M_{\theta }^{\left(i\right)},M_{x\theta }^{\left(i\right)}\right\} \end{array}$$

and $S^{\left(i\right)}$ is a stiffness matrix defined by

(24) $$S^{\left(i\right)}=\left[\begin{array}{llllll} A_{11}^{\left(i\right)} & A_{12}^{\left(i\right)} & 0 & B_{11}^{\left(i\right)} & B_{12}^{\left(i\right)} & 0\\ A_{12}^{\left(i\right)} & A_{22}^{\left(i\right)} & 0 & B_{12}^{\left(i\right)} & B_{22}^{\left(i\right)} & 0\\ 0 & 0 & A_{66}^{\left(i\right)} & 0 & 0 & B_{66}^{\left(i\right)}\\ B_{11}^{\left(i\right)} & B_{12}^{\left(i\right)} & 0 & D_{11}^{\left(i\right)} & D_{12}^{\left(i\right)} & 0\\ B_{12}^{\left(i\right)} & B_{22}^{\left(i\right)} & 0 & D_{12}^{\left(i\right)} & D_{22}^{\left(i\right)} & 0\\ 0 & 0 & B_{66}^{\left(i\right)} & 0 & 0 & D_{66}^{\left(i\right)} \end{array}\right]$$

in which $A_{pq}^{\left(i\right)}$, $B_{pq}^{\left(i\right)}$, and $D_{pq}^{\left(i\right)}$ (*p*, *q* = 1, 2, 6) are stretching, coupling, and bending stiffness coefficients and can be expressed as

(25) $$\left[\begin{array}{c} A_{pq}^{\left(i\right)}\\ B_{pq}^{\left(i\right)}\\ D_{pq}^{\left(i\right)} \end{array}\right]=\int_{-\frac{h}{2}}^{\frac{h}{2}}Q_{pq}^{\left(i\right)}\left[\begin{array}{c} 1\\ z\\ z^{2} \end{array}\right]\;dz$$

The strain energy and kinetic energy of the GPLRP, two-cylindrical-panel system can be written as

(26) $$U=\frac{1}{2}\sum_{i=1}^{2}\int_{0}^{L}\int_{-\theta_{0}/2}^{\theta_{0}/2}\varepsilon_{\left(i\right)}^{T}\cdot S^{\left(i\right)}\cdot \varepsilon_{\left(i\right)}Rd\theta_{i}dx_{i}$$

(27) $$T=\frac{1}{2}\sum_{i=1}^{2}\int_{0}^{L}\int_{-\theta_{0}/2}^{\theta_{0}/2}I_{\left(i\right)}\left[{\left(\frac{\partial u_{i}}{\partial t}\right)}^{2}+{\left(\frac{\partial v_{i}}{\partial t}\right)}^{2}+{\left(\frac{\partial w_{i}}{\partial t}\right)}^{2}\right]Rd\theta_{i}dx_{i}$$

where

(28) $$I_{\left(i\right)}=\int_{-\frac{h}{2}}^{\frac{h}{2}}\rho^{{}_{\left(i\right)}}(z)dz$$

The potential energy stored in the polymer matrix between the two-cylindrical-panel system can be written as

(29) $$U_{S}=\frac{1}{2}\int_{0}^{L}\int_{-\theta_{0}/2}^{\theta_{0}/2}K{\left(w_{1}-w_{2}\right)}^{2}Rd\theta_{i}dx_{i}$$

where *K* is the stiffness of Winkler springs.

For generality and convenience, the dimensionless parameters ${\bar{x}}_{i}$ and ${\bar{\theta }}_{i}$ are introduced

(30) $${\bar{x}}_{i}=\frac{2x_{i}}{L}-1,{\bar{\theta }}_{i}=\frac{2\theta_{i}}{\theta_{0}}$$

Substituting Equation (30) into Equations (16) and (17) yields

(31) $$\varepsilon_{1}^{\left(i\right)}=\frac{2}{L}\frac{\partial u_{i}}{\partial {\bar{x}}_{i}},\varepsilon_{2}^{\left(i\right)}=\frac{1}{R}\left(\frac{2}{\theta_{0}}\frac{\partial v_{i}}{\partial {\bar{\theta }}_{i}}+w_{i}\right),\gamma_{12}^{\left(i\right)}=\frac{2}{L}\frac{\partial v_{i}}{\partial {\bar{x}}_{i}}+\frac{1}{R}\frac{2}{\theta_{0}}\frac{\partial u_{i}}{\partial {\bar{\theta }}_{i}}$$

(32) $$\varphi_{1}^{\left(i\right)}=-\frac{4}{L^{2}}\frac{\partial^{2}w_{i}}{\partial {\bar{x}}_{i}{}^{2}},\;\;\varphi_{2}^{\left(i\right)}=-\frac{1}{R^{2}}\left(\frac{4}{\theta_{0}{}^{2}}\frac{\partial^{2}w_{i}}{\partial {\bar{\theta }}_{i}{}^{2}}-\frac{2}{\theta_{0}}\frac{\partial v_{i}}{\partial {\bar{\theta }}_{i}}\right),\;\varphi_{12}^{\left(i\right)}=-\frac{1}{R}\left(\frac{4}{L\theta_{0}}\frac{\partial^{2}w_{i}}{\partial {\bar{x}}_{i}\partial {\bar{\theta }}_{i}}-\frac{2}{L}\frac{\partial v_{i}}{\partial {\bar{x}}_{i}}\right)$$

By separating variables, the admissible displacement functions of the *i*th GPLRP, cylindrical panel can be given by

(33) $$\begin{array}{l} u_{i}(x_{i},\theta_{i},t)=U_{i}({\bar{x}}_{i},{\bar{\theta }}_{i})\sin(\omega t)\\ v_{i}(x_{i},\theta_{i},t)=V_{i}({\bar{x}}_{i},{\bar{\theta }}_{i})\sin(\omega t)\\ w_{i}(x_{i},\theta_{i},t)=W_{i}({\bar{x}}_{i},{\bar{\theta }}_{i})\sin(\omega t) \end{array}$$

where *t* is the time variable; the *ω* is the natural angular frequency; $U_{i}({\bar{x}}_{i},{\bar{\theta }}_{i})$;$V_{i}({\bar{x}}_{i},{\bar{\theta }}_{i})\;\;\;$ and $W_{i}({\bar{x}}_{i},{\bar{\theta }}_{i})$ are the displacement amplitude functions of the *i*th GPLRP, cylindrical panel.

The displacement amplitude functions of the *i*th GPLRP, cylindrical panel expressed by the Chebyshev polynomials can be given as [41]

(34) $$\begin{array}{l} U_{i}({\bar{x}}_{i},{\bar{\theta }}_{i})=f^{u}({\bar{x}}_{i},{\bar{\theta }}_{i})\sum_{m=1}^{M}\sum_{n=1}^{N}U_{mn}^{\left(i\right)}P_{m}({\bar{x}}_{i})P_{n}({\bar{\theta }}_{i})\\ V_{i}({\bar{x}}_{i},{\bar{\theta }}_{i})\;\;\;=f^{v}({\bar{x}}_{i},{\bar{\theta }}_{i})\sum_{m=1}^{M}\sum_{n=1}^{N}V_{mn}^{\left(i\right)}P_{m}({\bar{x}}_{i})P_{n}({\bar{\theta }}_{i})\\ W_{i}({\bar{x}}_{i},{\bar{\theta }}_{i})=f^{w}({\bar{x}}_{i},{\bar{\theta }}_{i})\sum_{m=1}^{M}\sum_{n=1}^{N}W_{mn}^{\left(i\right)}P_{m}({\bar{x}}_{i})P_{n}({\bar{\theta }}_{i}) \end{array}$$

where $U_{mn}^{\left(i\right)}$, $V_{mn}^{\left(i\right)}$, and $W_{mn}^{\left(i\right)}$ are the unknown coefficients; *M* and *N* are the truncation numbers of Chebyshev polynomials. $P_{m}({\bar{x}}_{i})$ and $P_{n}({\bar{\theta }}_{i})$ are the *m*th and *n*th Chebyshev polynomials of the first kind, which can be written as [51]

(35) $$P_{m}({\bar{x}}_{i})=\cos[(m-1)\arccos({\bar{x}}_{i})],P_{n}({\bar{\theta }}_{i})=\cos[(n-1)\arccos({\bar{\theta }}_{i})]$$

where $P_{m}({\bar{x}}_{i})$ and $P_{n}({\bar{\theta }}_{i})$ are a set of orthogonal polynomials in the interval [−1,1].

In addition, in Equation (34), functions $f^{\alpha }({\bar{x}}_{i},{\bar{\theta }}_{i})$, *α* = *u*, *v*, *w* are the auxiliary functions related to boundary conditions.

(36) $$f^{\alpha }({\bar{x}}_{i},{\bar{\theta }}_{i})={\left(1+{\bar{x}}_{i}\right)}^{p}{\left(1-{\bar{x}}_{i}\right)}^{q}{\left(1+\bar{\theta }\right)}^{r}{\left(1-\bar{\theta }\right)}^{s}$$

in which *p*, *q*, *r*, and *s* are equal to zero, one, or two, for different boundary conditions. For instance, in the GPLRP, two-cylindrical-panel system with clamped boundaries (C) on *x* = 0 and *x* = *L*, free (F) at *θ* = −*θ*_0_/2, and simply supported (S) at *θ* = *θ*_0_/2, auxiliary functions can be written as

(37) $$\begin{array}{l} f^{u}({\bar{x}}_{i},{\bar{\theta }}_{i})={\left(1+{\bar{x}}_{i}\right)}^{1}{\left(1-{\bar{x}}_{i}\right)}^{1}{\left(1+{\bar{\theta }}_{i}\right)}^{0}{\left(1-{\bar{\theta }}_{i}\right)}^{1}\\ f^{v}({\bar{x}}_{i},{\bar{\theta }}_{i})={\left(1+{\bar{x}}_{i}\right)}^{1}{\left(1-{\bar{x}}_{i}\right)}^{1}{\left(1+{\bar{\theta }}_{i}\right)}^{0}{\left(1-{\bar{\theta }}_{i}\right)}^{0}\\ f^{w}({\bar{x}}_{i},{\bar{\theta }}_{i})={\left(1+{\bar{x}}_{i}\right)}^{2}{\left(1-{\bar{x}}_{i}\right)}^{2}{\left(1+{\bar{\theta }}_{i}\right)}^{0}{\left(1-{\bar{\theta }}_{i}\right)}^{1} \end{array}$$

The strain energy of the GPLRP, two-cylindrical-panel system can be obtained by substituting Equations (24), (31)–(33) into (26):

(38) $$\begin{array}{ll} U= & \sum_{i=1}^{2}\int_{-1}^{1}\int_{-1}^{1}{\left[\sin(\omega \;t)\right]}^{2}\{\frac{A_{22}^{\left(i\right)}L\theta_{0}W_{i}{}^{2}}{8R}+\frac{A_{66}^{\left(i\right)}L}{2R\theta_{0}}{\left(\frac{\partial U_{i}}{\partial {\bar{\theta }}_{i}}\right)}^{2}+\frac{B_{22}^{\left(i\right)}LW_{i}}{2R^{2}}\frac{\partial V_{i}}{\partial {\bar{\theta }}_{i}}+\frac{A_{22}^{\left(i\right)}LW_{i}}{2R}\frac{\partial V_{i}}{\partial {\bar{\theta }}_{i}}\\ & +\frac{D_{22}^{\left(i\right)}L}{2R^{3}\theta_{0}}{\left(\frac{\partial V_{i}}{\partial {\bar{\theta }}_{i}}\right)}^{2}+\frac{B_{22}^{\left(i\right)}L}{R^{2}\theta_{0}}{\left(\frac{\partial V_{i}}{\partial {\bar{\theta }}_{i}}\right)}^{2}+\frac{A_{22}^{\left(i\right)}L}{2R\theta_{0}}{\left(\frac{\partial V_{i}}{\partial {\bar{\theta }}_{i}}\right)}^{2}-\frac{B_{22}^{\left(i\right)}LW_{i}}{R^{2}\theta_{0}}\frac{\partial^{2}W_{i}}{\partial {\bar{\theta }}_{i}{}^{2}}-\frac{2D_{22}^{\left(i\right)}L}{R^{3}\theta_{0}{}^{2}}\frac{\partial V_{i}}{\partial {\bar{\theta }}_{i}}\frac{\partial^{2}W_{i}}{\partial {\bar{\theta }}_{i}{}^{2}}\\ & -\frac{2B_{22}^{\left(i\right)}L}{R^{2}\theta_{0}{}^{2}}\frac{\partial V_{i}}{\partial {\bar{\theta }}_{i}}\frac{\partial^{2}W_{i}}{\partial {\bar{\theta }}_{i}{}^{2}}+\frac{2D_{22}^{\left(i\right)}L}{R^{3}\theta_{0}{}^{3}}{\left(\frac{\partial^{2}W_{i}}{\partial {\bar{\theta }}_{i}{}^{2}}\right)}^{2}+\frac{A_{12}^{\left(i\right)}\theta_{0}W_{i}}{2}\frac{\partial U_{i}}{\partial {\bar{x}}_{i}}+A_{12}^{\left(i\right)}\frac{\partial V_{i}}{\partial {\bar{\theta }}_{i}}\frac{\partial U_{i}}{\partial {\bar{x}}_{i}}\\ & +\frac{B_{12}^{\left(i\right)}}{R}\frac{\partial V_{i}}{\partial {\bar{\theta }}_{i}}\frac{\partial U_{i}}{\partial {\bar{x}}_{i}}-\frac{2B_{12}^{\left(i\right)}}{R\theta_{0}}\frac{\partial^{2}W_{i}}{\partial {\bar{\theta }}_{i}{}^{2}}\frac{\partial U_{i}}{\partial {\bar{x}}_{i}}+\frac{A_{11}^{\left(i\right)}R\theta_{0}}{2L}{\left(\frac{\partial U_{i}}{\partial {\bar{x}}_{i}}\right)}^{2}+A_{66}^{\left(i\right)}\frac{\partial U_{i}}{\partial {\bar{\theta }}_{i}}\frac{\partial V_{i}}{\partial {\bar{x}}_{i}}\\ & +\frac{2B_{66}^{\left(i\right)}}{R}\frac{\partial U_{i}}{\partial {\bar{\theta }}_{i}}\frac{\partial V_{i}}{\partial {\bar{x}}_{i}}+\frac{2B_{66}^{\left(i\right)}\theta_{0}}{L}{\left(\frac{\partial V_{i}}{\partial {\bar{x}}_{i}}\right)}^{2}+\frac{2D_{66}^{\left(i\right)}\theta_{0}}{LR}{\left(\frac{\partial V_{i}}{\partial {\bar{x}}_{i}}\right)}^{2}+\frac{A_{66}^{\left(i\right)}R\theta_{0}}{2L}{\left(\frac{\partial V_{i}}{\partial {\bar{x}}_{i}}\right)}^{2}\\ & -\frac{4B_{66}^{\left(i\right)}}{R\theta_{0}}\frac{\partial U_{i}}{\partial {\bar{\theta }}_{i}}\frac{\partial^{2}W_{i}}{\partial {\bar{x}}_{i}\partial {\bar{\theta }}_{i}}-\frac{4B_{66}^{\left(i\right)}}{L}\frac{\partial V_{i}}{\partial {\bar{x}}_{i}}\frac{\partial^{2}W_{i}}{\partial {\bar{x}}_{i}\partial {\bar{\theta }}_{i}}-\frac{8D_{66}^{\left(i\right)}}{LR}\frac{\partial V_{i}}{\partial {\bar{x}}_{i}}\frac{\partial^{2}W_{i}}{\partial {\bar{x}}_{i}\partial {\bar{\theta }}_{i}}+\frac{8D_{66}^{\left(i\right)}}{LR\theta_{0}}{\left(\frac{\partial^{2}W_{i}}{\partial {\bar{x}}_{i}\partial {\bar{\theta }}_{i}}\right)}^{2}\\ & -\frac{B_{12}^{\left(i\right)}\theta_{0}W_{i}}{L}\frac{\partial^{2}W_{i}}{\partial {\bar{x}}_{i}{}^{2}}-\frac{2B_{12}^{\left(i\right)}}{L}\frac{\partial V_{i}}{\partial {\bar{\theta }}_{i}}\frac{\partial^{2}W_{i}}{\partial {\bar{x}}_{i}{}^{2}}-\frac{2D_{12}^{\left(i\right)}}{LR}\frac{\partial V_{i}}{\partial {\bar{\theta }}_{i}}\frac{\partial^{2}W_{i}}{\partial {\bar{x}}_{i}{}^{2}}+\frac{4D_{12}^{\left(i\right)}}{LR\theta_{0}}\frac{\partial^{2}W_{i}}{\partial {\bar{\theta }}_{i}{}^{2}}\frac{\partial^{2}W_{i}}{\partial {\bar{x}}_{i}{}^{2}}\\ & -\frac{2B_{11}^{\left(i\right)}R\theta_{0}}{L^{2}}\frac{\partial U_{i}}{\partial {\bar{x}}_{i}}\frac{\partial^{2}W_{i}}{\partial {\bar{x}}_{i}{}^{2}}+\frac{2D_{11}^{\left(i\right)}R\theta_{0}}{L^{3}}{\left(\frac{\partial^{2}W_{i}}{\partial {\bar{x}}_{i}{}^{2}}\right)}^{2}\}d{\bar{\theta }}_{i}d{\bar{x}}_{i} \end{array}$$

The kinetic energy of the GPLRP, two-cylindrical-panel system can be obtained by substituting Equation (33) into Equation (27):

(39) $$T=\sum_{i=1}^{2}\int_{-1}^{1}\int_{-1}^{1}\frac{1}{8}LR\theta_{0}\omega^{2}{\left[\cos(\omega t)\right]}^{2}\left[I_{\left(i\right)}\left(U_{i}{}^{2}+V_{i}{}^{2}+W_{i}{}^{2}\right)\right]d{\bar{\theta }}_{i}d{\bar{x}}_{i}$$

The potential energy of the polymer matrix can be acquired by substituting Equation (33) into Equation (29):

(40) $$U_{S}=\int_{-1}^{1}\int_{-1}^{1}\frac{LRK\theta_{0}}{8}{\left[\sin(\omega t)\right]}^{2}{\left(W_{1}-W_{2}\right)}^{2}d{\bar{\theta }}_{i}d{\bar{x}}_{i}$$

To determine the solutions, the Rayleigh–Ritz method is employed. The Lagrangian energy function Π of the GPLRP, two-cylindrical-panel system can be expressed as

(41) $$\Pi =U_{\max}+U_{S\max}-T_{\max}$$

in which *U*_max_ and *T*_max_ are the maximum strain energy and maximum kinetic energy of the GPLRP, two-cylindrical-panel system; *U*_Smax_ is the maximum potential energy of the polymer matrix.

Then, the Lagrangian energy function Π is minimized with respect to the unknown coefficient, yielding

(42) $$\frac{\partial \prod }{\partial U_{mn}^{\left(i\right)}}=0,\frac{\partial \prod }{\partial V_{mn}^{\left(i\right)}}=0,\frac{\partial \prod }{\partial W_{mn}^{\left(i\right)}}=0,(i=1,2;m=1,\ldots ,M;n=1,\ldots ,N)$$

The eigenvalue equations derived from Equation (42) can be summarized in the matrix form as

(43) $$\left(K-\omega^{2}M\right)X=0$$

in which **M** and **K** denote the mass and stiffness matrices, respectively. The explicit forms of the modal vector **X** can be written as follows:

(44) $$X={\left\{X^{\left(1\right)},X^{\left(2\right)}\right\}}^{T}$$

in which

(45) $$X^{\left(i\right)}=\left\{U_{11}^{\left(i\right)},\ldots ,U_{MN}^{\left(i\right)},V_{11}^{\left(i\right)},\ldots ,V_{MN}^{\left(i\right)},W_{11}^{\left(i\right)},\ldots ,W_{MN}^{\left(i\right)}\right\},(i=1,2)$$

For convenience, the dimensionless natural frequency of the GPLRP, two-cylindrical-panel system is defined by

(46) $$\bar{\omega }=\omega R\sqrt{\rho_{m}/E_{m}}$$

## 3. Model Validation and Convergence Analysis

To prove the validity of the present formulations, the dimensionless fundamental frequency $\bar{\omega }=\omega L\sqrt{\rho_{m}(1-\mu_{m}^{2})/E_{m}}$ of a single GPLRP plate under simply supported boundary conditions is compared with the results in the existing literature [41]. The material parameters used are consistent with the literature [41]. The geometric parameters of a single GPLRP cylindrical panel are as follows: *h* = 0.1 m, *L*/*S* = 1, *L*/*h* = 20, *W*_GPL_ = 1.0%, *e*_1_ = 0.5, *i* = 1, *K* = 0, and *R* = ∞. As shown in Table 1, it can be clearly seen that the results of this paper are well consistent with the literature [41].

To further clarify the correctness of the derivation of this study, Table 2 shows the comparison of the natural angular frequencies *ω* (rad/s) in this article with existing literature for the double-plate system under the simply supported boundary condition [26]. The geometric and material parameters are as follows: *L* = 1 m, *S* = 2 m, *E* = 1 × 10^10^ N/m^2^, *h* = 1 × 10^−2^ m, *i* = 1, 2, *K* = 6 × 10^4^ N/m^3^, *μ =* 0.3, *ρ =* 5 × 10^3^ kg/m^3^, and *R* = ∞. As can be observed in Table 2, very good agreement was obtained in this comparison study. The above comparisons with the open literature are sufficient to prove the accuracy of the derivation in this paper.

In this study, the Rayleigh–Ritz method was applied to solve the natural frequencies of the GPLRP, two-cylindrical-panel system. The accuracy of this method depends on the truncation numbers (*M*, *N*) of the Chebyshev polynomials. Therefore, it is necessary to discuss the influence of truncation orders *M* and *N* on the calculation results. The simulation parameters are given in Section 4. As shown in Table 3, when the truncation numbers *M* = *N* = 11, the dimensionless natural frequencies $\bar{\omega }=\omega R\sqrt{\rho_{m}/E_{m}}$ obtained good convergence. Therefore, the truncation numbers *M* and *N* were set to 11 in the following study.

## 4. Results and Discussion

In this section, the material and geometric parameters of the GPLRP, two-cylindrical-panel system in this paper used are as follows: *E*_m_ = 130 GPa, *ρ*_m_ = 8960 kg/m^3^, *μ*_m_ = 0.34, *E*_GPL_ = 1.01 Tpa, *ρ*_GPL_ *=* 1062.5 kg/m^3^*,* *μ*_GPL_ = 0.186, *t*_GPL_ = 1.5 nm, *l*_GPL_ = 2.5 μm, *w*_GPL_ = 1.5 μm, *W*_GPL_ = 1.0%, *e*_1_ = 0.5, *R* = 1 m, *h* = 0.01*R*, *L* = *R*, *θ*_0_ = π/6, and *K* = 1 × 10^14^ N/m^3^.

**Figure 4:**
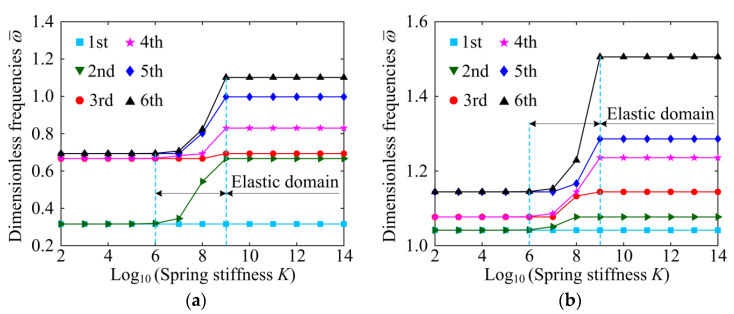
The influences of spring stiffness on first six dimensionless natural frequencies of the GPLRP, two-cylindrical-panel system (Porosity-I, GPL A, *e*_1_ = 0.5). (**a**) SSSS; (**b**) CCCC.

Figure 4 shows the variation un dimensionless natural frequencies of the system with the spring stiffness *K*, considering different boundary conditions. An interesting phenomenon is that the variation of spring stiffness has no influence on the first-order natural frequency of the system. In addition, there is an elastic domain for other natural frequencies except for the first-order natural frequency, in which the natural frequency increases rapidly with the increase in the spring stiffness *K*. This finding is further confirmed by the results in Table 4. The reason for this phenomenon is that the natural frequency of the GPLRP, two-cylindrical-panel system can be divided into two parts, one of which is the same as the natural frequency of a single GPLRP, cylindrical panel, and the other is identical to the natural frequency of a single GPLRP, cylindrical panel on an elastic foundation with the stiffness 2*K* [26]. Therefore, except for the first-order natural frequency, other natural frequencies can be adjusted by adjusting the value of the spring stiffness to obtain the desired natural frequency. This means that the stiffness of the system can be changed by adjusting the spring stiffness. For the sake of brevity, only the fundamental frequency of the system is discussed below.

**Figure 5:**
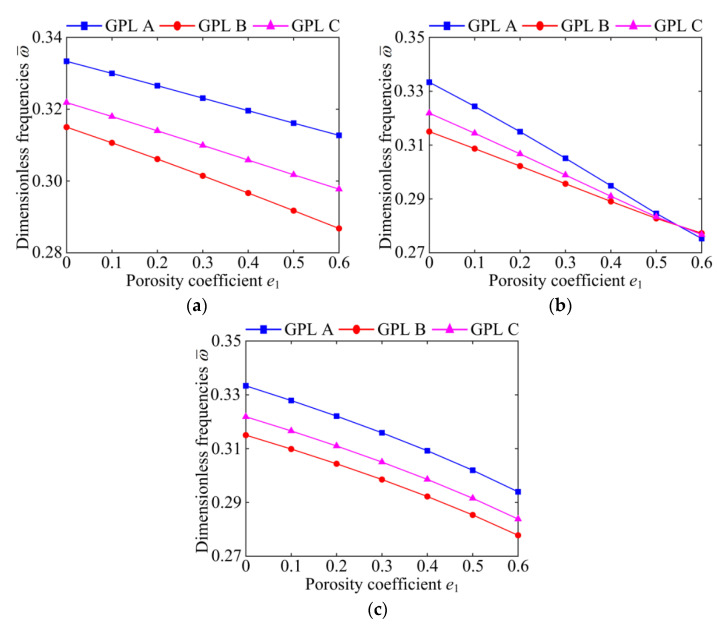
The influences of porosity coefficient on dimensionless fundamental frequencies of the GPLRP, two-cylindrical-panel system (SSSS). (**a**) Porosity-I; (**b**) Porosity-II; (**c**) Porosity-III.

**Figure 6:**
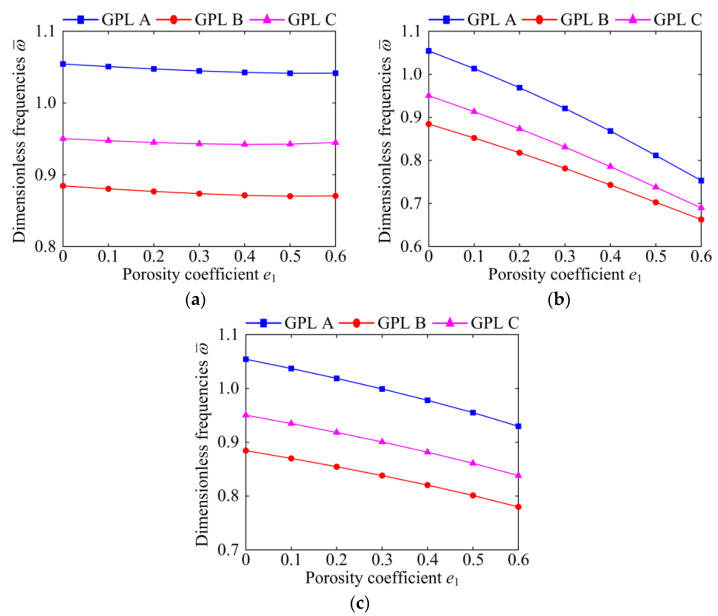
The influences of porosity coefficient on dimensionless fundamental frequencies of the GPLRP, two-cylindrical-panel system (CCCC). (**a**) Porosity-I; (**b**) Porosity-II; (**c**) Porosity-III.

Figure 5 and Figure 6 demonstrate the effects of the porosity coefficient on dimensionless fundamental frequencies of the system for specific porosity distributions and boundary conditions. It can be clearly seen that the natural frequency of the system decreases significantly with the increase in the porosity coefficient, except for Porosity-I under the CCCC boundary condition. This indicates that in most cases, the greater the porosity coefficient *e*_1_, the weaker the stiffness of the system. Then, comparing Figure 5a and Figure 6a, it can also be seen that the natural frequency of the system under the SSSS boundary condition decreases rapidly with the increase in the porosity coefficient. However, the natural frequency of the system under the CCCC boundary condition changes slightly with the increase in the porosity coefficient, which indicates that the combined influence of the porosity coefficient and GPL distribution on the natural frequency is related to the boundary conditions. It is also notable that when *e*_1_ ≤ 0.5, for all porosity distributions, GPL A possesses the largest natural frequency and GPL B possesses the smallest natural frequency. However, when *e*_1_ > 0.5, GPL A does not always possess the largest natural frequency; for example, see Porosity-II under the SSSS boundary condition. It can be concluded that the effect of the porosity coefficient on the natural frequency of the system is associated with the porosity distributions, GPL distribution patterns, and boundary conditions.

**Figure 7:**
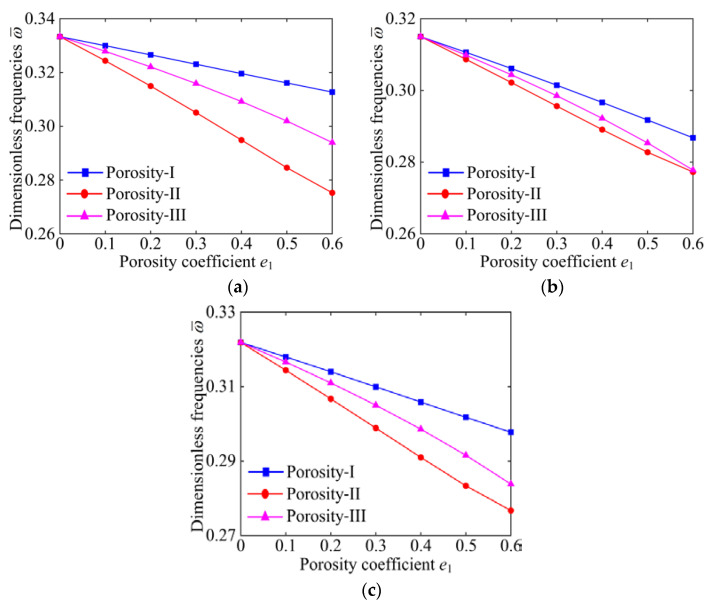
The influences of porosity coefficient on dimensionless fundamental frequencies of the GPLRP, two-cylindrical-panel system (SSSS). (**a**) GPL A; (**b**) GPL B; (**c**) GPL C.

**Figure 8:**
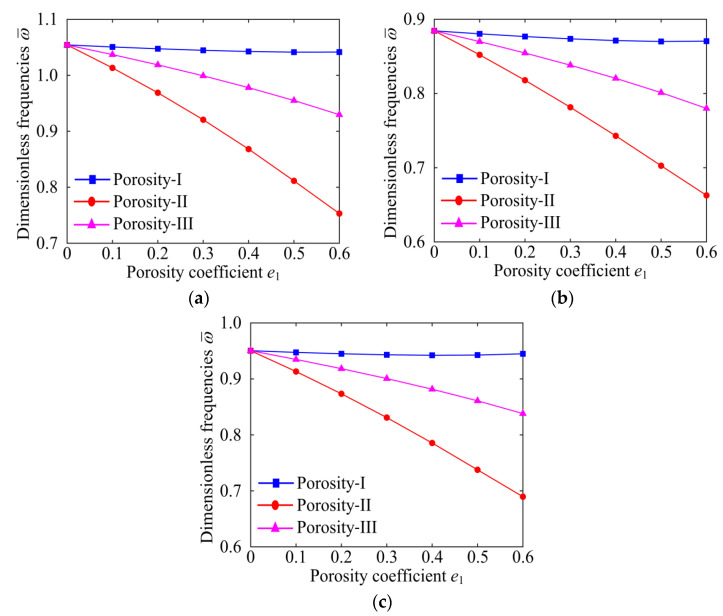
The influences of porosity coefficient on dimensionless fundamental frequencies of the GPLRP, two-cylindrical-panel system (CCCC). (**a**) GPL A; (**b**) GPL B; (**c**) GPL C.

To explain the effect of porosity distribution more clearly, Figure 7 and Figure 8 indicate the dimensionless fundamental frequencies versus the porosity coefficient for different porosity distributions with specific GPL distribution patterns and boundary conditions. As can be seen in Figure 7 and Figure 8, for all kinds of porosity distributions, Porosity-I possesses the largest natural frequency. This indicates that Porosity-I can significantly enhance the stiffness of the system. It is worth noting that the natural frequency of the system decreases significantly with the increase in the porosity coefficient, except for the Porosity-I under the CCCC boundary condition. This further confirms that the effect of the porosity distributions on the natural frequency of the system is associated with the boundary conditions. From the above results, it can be confirmed that the porosity coefficient, porosity distribution, GPL distribution pattern, and the boundary condition interact with each other and have coupled effect on the free vibration characteristics of the GPLRP, two-cylindrical-panel system.

**Figure 9:**
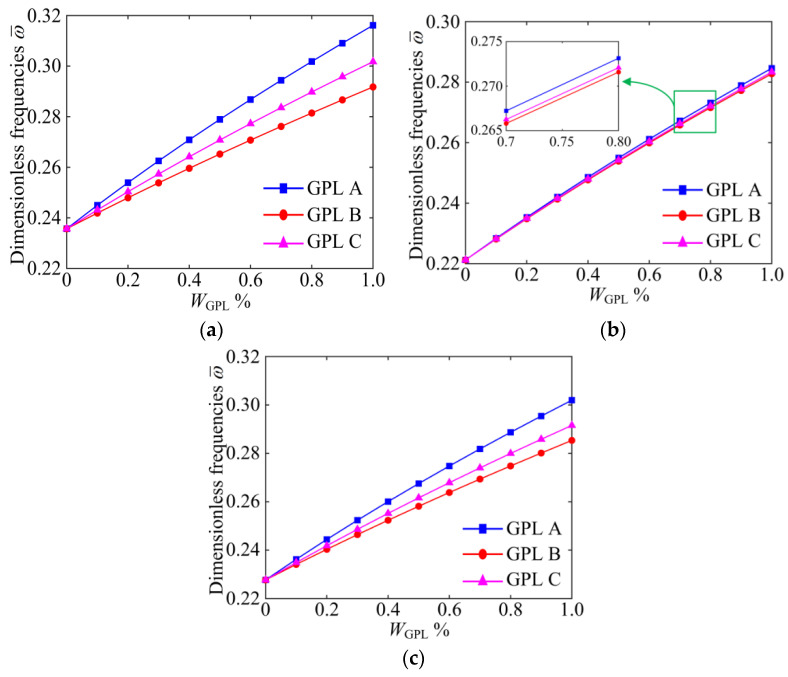
The influences of GPL weight fraction on dimensionless fundamental frequencies of the GPLRP, two-cylindrical-panel system (SSSS). (**a**) Porosity-I; (**b**) Porosity-II; (**c**) Porosity-III.

Figure 9 reveals the variations in dimensionless fundamental frequencies with GPL weight fraction *W*_GPL_ for different porosity distributions and GPL distribution patterns. It is clearly shown that the natural frequency increases significantly with the increase in the weight fraction *W*_GPL_ for different porosity distributions and GPL distribution patterns. This means that the GPL filling material can significantly improve the effective stiffness of the system. As shown in Figure 9b, it was interesting to find that all GPL distribution patterns in Porosity-II have almost the same increase in efficiency with the increase in the GPL weight fraction *W*_GPL_. This indicates that the porosity distributions play a dominant role, and it also shows that choosing an appropriate porosity distribution is of great significance to improving the stiffness of the system. This means that the strength of the system can be improved by choosing the appropriate porosity distribution.

## 5. Conclusions

This study proposed a dynamic model of the GPLRP, two-cylindrical-panel system with general boundary conditions based on Love’s shell theory to study the free vibration characteristics of the system. The Rayleigh–Ritz method is used to calculate the solutions. From the results, some valuable conclusions can be drawn as follows:

(1) The variation in the stiffness of the Winkler springs in the GPLRP, two-cylindrical-panel system has no effect on the first-order natural frequency, but other natural frequencies can be adjusted by controlling the value of the spring stiffness to obtain the desired natural frequency.
(2) The porosity coefficient, porosity distribution, GPL distribution pattern, and boundary condition interact with each other and have a coupled influence on the vibration characteristics of the GPLRP, two-cylindrical-panel system.
(3) Increasing the proportion of the GPL filling material can significantly improve the stiffness of the GPLRP, two-cylindrical-panel system, thereby increasing the natural frequency of the system.

## Figures and Tables

**Table 1:** Comparison of the dimensionless fundamental frequency of a single GPLRP plate.

Porosity Distributions	GPL Distributions	Present	Yang et al. [41]
	GPL A	0.4038	0.3958
Porosity-I	GPL B	0.3333	0.3293
	GPL C	0.3633	0.3574
	GPL A	0.3089	0.3072
Porosity-II	GPL B	0.2611	0.2601
	GPL C	0.2766	0.2754
	GPL A	0.3686	0.3627
Porosity-III	GPL B	0.3043	0.3014
	GPL C	0.3294	0.3252

**Table 2:** Comparison of the natural angular frequencies of the double-plate system.

Modes	Present	Oniszczuk [26]
1	52.8	52.8
2	72.0	72.0
3	84.5	84.5
4	97.7	97.7
5	137.3	137.3
6	145.8	145.8

**Table 3:** Convergence of the first six dimensionless natural frequencies of the GPLRP, two-cylindrical-panel system (Porosity-I, GPL A, *e*_1_ = 0.5).

	Mode	(*M*, *N*)						
		(7, 7)	(8, 8)	(9, 9)	(10, 10)	(11, 11)	(12, 12)	(13, 13)
	1	0.31612	0.31612	0.31612	0.31612	0.31612	0.31612	0.31612
	2	0.66671	0.66671	0.66667	0.66667	0.66667	0.66667	0.66667
	3	0.69330	0.69330	0.69330	0.69330	0.69330	0.69330	0.69330
SSSS	4	0.83013	0.83013	0.83009	0.83009	0.83009	0.83009	0.83009
	5	0.99756	0.99751	0.99746	0.99746	0.99746	0.99746	0.99746
	6	1.10223	1.10134	1.10126	1.10125	1.10125	1.10125	1.10125
	1	1.04144	1.04143	1.04143	1.04143	1.04143	1.04143	1.04143
	2	1.07692	1.07691	1.07690	1.07690	1.07690	1.07690	1.07690
CCCC	3	1.14413	1.14411	1.14411	1.14411	1.14411	1.14411	1.14411
	4	1.23574	1.23571	1.23570	1.23570	1.23570	1.23570	1.23570
	5	1.28614	1.28609	1.28607	1.28607	1.28607	1.28607	1.28607
	6	1.50574	1.50572	1.50569	1.50569	1.50568	1.50568	1.50568

**Table 4:** The influences of spring stiffness on dimensionless natural frequencies of the GPLRP, two-cylindrical-panel system (Porosity-I, GPL A, *e*_1_ = 0.5).

	Mode	Spring Stiffness *K*						
		10^2^	10^4^	10^6^	10^8^	10^10^	10^12^	10^14^
SSSS	1	0.31612	0.31612	0.31612	0.31612	0.31612	0.31612	0.31612
	2	0.31612	0.31615	0.31920	0.54342	0.66667	0.66667	0.66667
	3	0.66667	0.66667	0.66667	0.66667	0.69330	0.69330	0.69330
	4	0.66667	0.66668	0.66816	0.69330	0.83009	0.83009	0.83009
	5	0.69330	0.69330	0.69330	0.80272	0.99746	0.99746	0.99746
	6	0.69330	0.69331	0.69472	0.82358	1.10125	1.10125	1.10125
CCCC	1	1.04143	1.04143	1.04143	1.04143	1.04143	1.04143	1.04143
	2	1.04143	1.04144	1.04238	1.07690	1.07690	1.07690	1.07690
	3	1.07690	1.07690	1.07690	1.13267	1.14411	1.14411	1.14411
	4	1.07690	1.07691	1.07784	1.14411	1.23570	1.23570	1.23570
	5	1.14411	1.14411	1.14411	1.16643	1.28607	1.28607	1.28607
	6	1.14411	1.14412	1.14498	1.22876	1.50568	1.50568	1.50568
